# Splice site proximity influences alternative exon definition

**DOI:** 10.1080/15476286.2022.2089478

**Published:** 2022-06-19

**Authors:** Francisco Carranza, Hossein Shenasa, Klemens J. Hertel

**Affiliations:** Department of Microbiology and Molecular Genetics, University of California Irvine, Irvine, California, USA

**Keywords:** Alternative splicing, exon definition, Intron-exon architecture, splice site selection, bioinformatics, molecular biology

## Abstract

Alternative splicing enables higher eukaryotes to expand mRNA diversity from a finite number of genes through highly combinatorial splice site selection mechanisms that are influenced by the sequence of competing splice sites, cis-regulatory elements binding trans-acting factors, the length of exons and introns harbouring alternative splice sites and RNA secondary structures at putative splice junctions. To test the hypothesis that the intron definition or exon definition modes of splice site recognition direct the selection of alternative splice patterns, we created a database of alternative splice site usage (ALTssDB). When alternative splice sites are embedded within short introns (intron definition), the 5′ and 3′ splice sites closest to each other across the intron preferentially pair, consistent with previous observations. However, when alternative splice sites are embedded within large flanking introns (exon definition), the 5′ and 3′ splice sites closest to each other across the exon are preferentially selected. Thus, alternative splicing decisions are influenced by the intron and exon definition modes of splice site recognition. The results demonstrate that the spliceosome pairs splice sites that are closest in proximity within the unit of initial splice site selection.

## Introduction

Pre-mRNA splicing is an essential step in eukaryotic gene expression that involves the excision of intronic sequences and the transesterification of exonic sequences by the spliceosome to generate protein coding mRNAs. Alternative exon inclusion is possible through a process known as alternative splicing. At least 95% of human genes undergo alternative splicing in response to cell cycle, developmental, tissue-specific or signalling cues. Alternative splicing increases proteomic diversity from a limited genome in a regulated fashion [1]. Thus, pre-mRNA splicing impacts gene expression [2].

The recognition of splice junctions by the spliceosome initiates the splicing reaction. The 5′ splice site (5′ss) is defined by a nine-nucleotide consensus sequence that spans the exon/intron junction at the 5′ end of each intron. The 3′ splice site (3′ss) includes three sequence elements found within an approximately 40 nucleotides (nts) stretch, upstream of the 3′ intron/exon junction. These include the intron/exon junction sequence, which contains the essential AG dinucleotide at the 3′ end of the intronic sequence, the polypyrimidine tract (PPT), a region containing 15–20 pyrimidines located upstream of the intron/exon junction and the branch point sequence, a highly degenerate sequence that contains a conserved adenosine located upstream of the PPT.

Exon recognition is a highly combinatorial process that is known to be influenced by many cis- and trans-acting features. These include splicing enhancers, silencers, RNA secondary structure, the intron-exon architecture and the sequence context of splice junctions [3–5]. The strength of splice sites is determined by how well they conform to consensus splice junction motifs that function in recruiting U1 snRNP to the 5′ss and U2AF to the 3′ss. Consensus similarity scores, derived from the modelling of short sequence motifs using the maximum-entropy principle (MaxEnt), define splice site strength numerically [6]. Splice sites are known to act synergistically and combined 5′ and 3′ss scores are a much better predictor for exon inclusion than either splice site score alone [7]. Importantly, the ability of an exon to undergo various forms of alternative splicing is heavily influenced by the strength of its splice sites [8].

**Figure 1.**
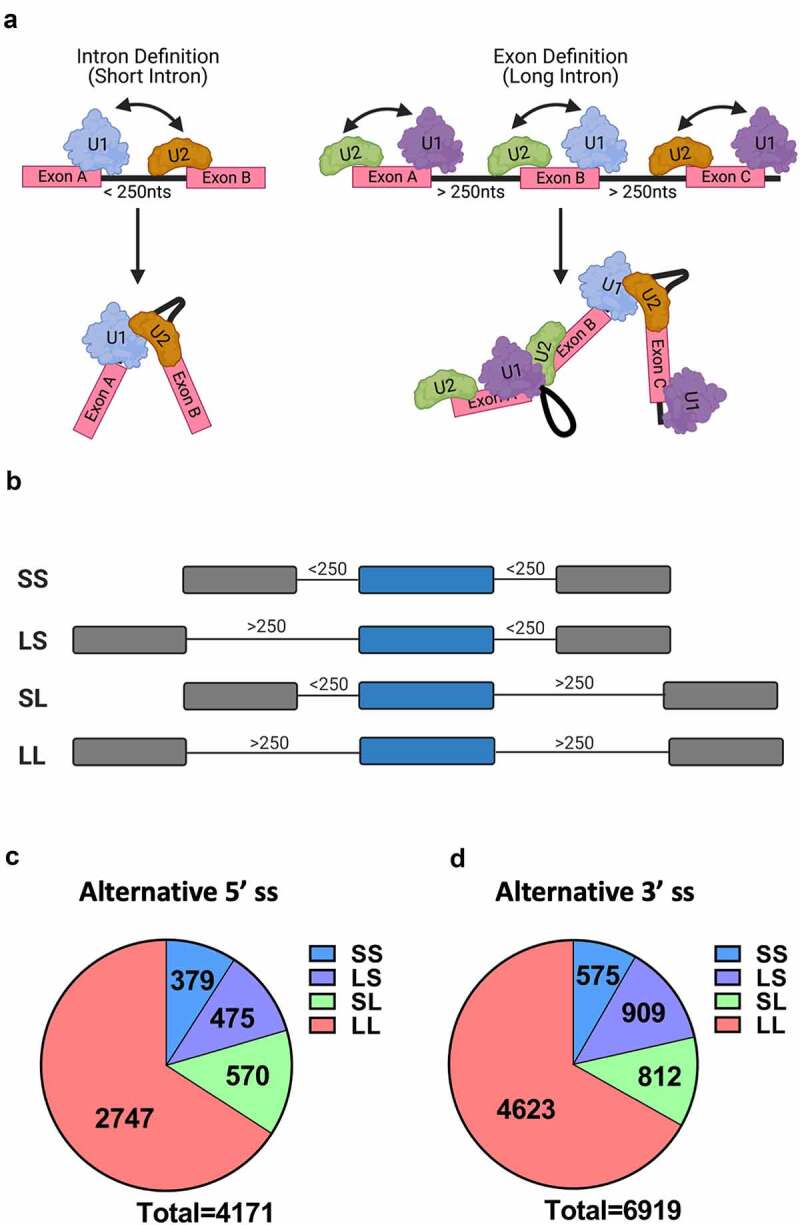
Gene architecture and database (a) The two proposed modes of splice site recognition. During intron definition splice sites are recognized across the intron (left). Under exon definition (right) splice sites are initially recognized across the exon, followed by splice site juxta-position. (b) ALTssDB categories of internal exons as defined by flanking intron size. S stands for short (less than 250 nts), L stands for long (greater than 250 nts). (c) and (d) Distribution of ALTssDB internal exon categories for alternative 5′ (c) and alternative 3′ (d) splice site events.

Another crucial factor in splice site selection is the genomic architecture [9–12]. The genomes in lower eukaryotes are characterized almost exclusively by the presence of short introns (<250 nts). By contrast, human genes harbour long introns, with >87% of introns longer than 250 nts [10]. This different genomic architecture has been shown to contribute significantly to the manner in which spliceosomal assembly occurs. The two proposed mechanisms through which splice sites are recognized are referred to as the exon or intron definition mode of splice site recognition (Figure 1(a)). During intron definition, the spliceosome assembles across the intron that will be excised. Under conditions that promote exon definition, initial splice site recognition is postulated to occur across the exon. This initial recognition is predicted to be followed by an additional splice site juxta-positioning step to induce intron excision. *In vitro* splicing and transfection experiments of designer minigenes demonstrated that the transition between intron and exon definition occurs at an intron length of approximately 250 nts [10]. Thus, splice sites that are flanked by large introns (>250 nts) are recognized through exon definition, while intron-defined splice sites are associated with small flanking introns (<250 nts). It is currently unknown how exon and intron definition influence alternative splice site selection.

Understanding the relationship between the splice site strength and intron-exon architecture splicing determinants has been a longstanding goal in deciphering the splicing code. The mechanisms utilized by the spliceosome to select the correct splice site in the presence of multiple nearby cryptic or alternative splice sites are still not completely understood. Differences in intron-exon architecture and splice site strength are known to be important in mediating alternative splice site selection [8]. A series of classical experiments demonstrated that the proximity between the 5′ and 3′ splice sites, across the intron, plays a crucial role in splice site preference [11]. Reed and Maniatis showed that the splice site closest to its intronic splicing partner was favoured over a distal competing splice site [11]. Thus, in the case of competing alternative 5′ splice sites, the downstream 5′ss was preferred because it was more proximal to the pairing 3′ss. Similarly, between competing 3′ splice sites, the upstream 3′ss was chosen. These observations suggest that in the absence of confounding factors, shorter distances between splice sites are favoured during intron-defined splicing. This may be because splice site pairing is more efficient across shorter distances. These experiments established a splice site selection proximity rule (for clarity referred to as the intron-centric proximity rule); however, it is unclear how dominant it is within the hierarchical nature of known splicing determinants.

In this study, we carried out computational analyses to assess the impact of the intron-centric proximity rule. We demonstrate that the intron-centric proximity rule is generally applicable for the intron definition mode of splice site definition. For the exon definition mode of splice site definition, we observe an exon-centric proximity rule that deviates from the classical intron-centric proximity rule. The 5′ and 3′ splice sites closest to each other across the exon are preferentially selected. Thus, when the unit of splice site definition is across the intron (intron definition), the 5′ and 3′ splice sites closest to each other across the intron preferentially pair. When the unit of splice site recognition is the exon (exon definition), the 5′ and 3′ splice sites closest to each other across the exon are preferentially selected. Our results provide evidence that alternative splicing decisions are influenced by the intron and exon definition modes of splice site recognition.

## Results

### The influence of intron-exon architecture on 5′ splice site selection

To determine the impact of the intron-exon architecture and splice site strength on splice site selection, we created a database of alternative splice sites (ALTssDB) using the Human Exon Splicing Event Database HEXEvent [13], the Intron DB [14] and GeneBase [15]. MaxEntScan, a computational tool, was used to assign splice site scores [6]. To minimize variability, we focused on competing alternative 5′ or 3′ splice site pairs of internal exons with only one alternative splice pattern. Thus, ALTssDB catalogs pairs of alternative 5′ splice sites competing for a common 3′ss or pairs of alternative 3′ splice sites competing for a common 5′ss. ALTssDB reports the location of the major splice site and its competing alternative 3′ or 5′ splice site, corresponding exon sizes, usage levels, splice site scores and flanking intron lengths. Using these filters, ALTssDB captures 4,171 human 5′ss competition events and 6,919 human 3′ss competition events (Figure 1(b-d)).

**Figure 2.**
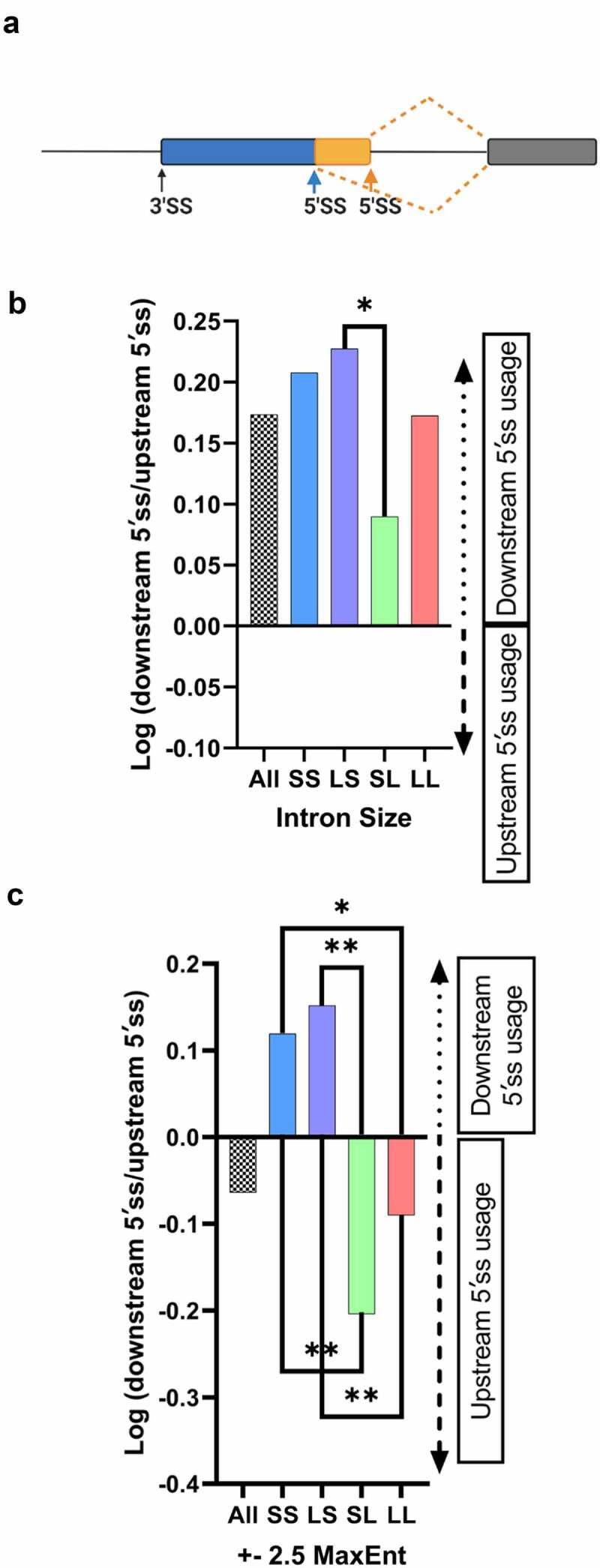
5′ ss selection preference for different internal exon categories. (a) Model depicting alternative 5′ss patterns. (b) Bar graph depicting the preference for downstream or upstream 5′ ss selection for different internal exon categories (sample size = 379 SS, 2,747 LL, 570 LS, 475 SL). A positive log ratio represents downstream 5′ ss preference, a negative log ratio represents upstream 5′ ss preference. (c) Splice site selection preference for alternative 5′ splicing events with near equal splice-site strength scores (∆ ± 2.5 MaxEnt, sample size = 88 SS, 725 LL, 104 LS, 143 SL), Fisher’s exact test was performed. (b, c), **p < 0.01, * p < 0.05.

We first tested whether the intron-centric proximity rule holds true when evaluating all alternative 5′ss events transcriptome-wide (Figure 2(a)). In agreement with the intron-centric proximity rule expectation that the downstream 5′ss should be selected over a competing upstream 5′ss, we observed a preference for downstream 5′ss selection in ~60% (2,497) of the alternative 5′ss splicing events (Figure 2(b), left bar).

To evaluate whether the ‘intron definition’ or ‘exon definition’ mode of splice site selection influence adherence to the intron-centric proximity rule, we parsed the 5′ss dataset into intron definition events (379 SS), exon definition events (2,747 LL), and hybrid events (570 LS, 475 SL) (Figure 1(b and c)). For the purpose of alternative 5′ss selection analysis, the hybrid architectural class LS was categorized as intron defined because the 5′ss is adjacent to a short intron and U1 snRNP binding to the 5′ss at the exon/intron junction initiates early spliceosome formation [16]. By analogy, the architectural class SL was considered exon defined because the 5′ss is contained within a long intron. Surprisingly, in all four intron architecture classes, the majority of events still displayed a preference for the downstream 5′ss, consistent with the intron-centric proximity rule, albeit to varying degrees (Figure 2(b)). For example, the downstream 5′ss is selected more frequently for intron definition events (represented by SS, LS) when compared to exon definition events (represented by LL, SL). These varying degrees of preference suggest that the intron definition mode of splice site selection adheres more stringently to the intron-centric proximity rule.

### The influence of intron-exon architecture on 5′ss selection in the absence of splice site strength differences

One important determinant that may mask the influence of splice site proximity is the difference in the splice site strength of competing splice sites. To determine the impact of splice site strength on alternative 5′ss selection, we compared the splice strength of the major 5′ss versus the alternative 5′ss. In 86% of the events evaluated the 5′ss with a higher predicted splice strength was the dominant 5′ss, irrespective of whether the exon was predicted to be recognized through exon definition (LL, SL) (85%) or intron definition (SS, LS) (90%) events. These results support the notion that splice site strength is a strong determinant in alternative 5′ss selection.

To determine how the exon and intron definition modes of splice site selection influence alternative splicing the impact of splice site strength differences was minimized computationally. This was achieved by isolating 5′ss competition events with near equal splice site scores (∆MaxEnt = ±2.5), resulting in 88 SS, 725 LL, 104 LS, and 143 SL events. Interestingly, when this splice site strength filter was applied, we observed that the upstream 5′ss is preferentially selected in 60% of competition events, inconsistent with the expectations of the intron-centric proximity rule (Figure 2(c), left bar). Strict intron definition events (SS category) display a downstream 5′ss selection preference, consistent with the intron-centric proximity rule, while strict exon definition events (LL) display a preference for the upstream 5′ splice site (Figure 2(c)). The upstream preference under exon definition is inconsistent with the intron-centric proximity rule but consistent with an exon-centric proximity rule. These biases are heightened in the hybrid categories SL (upstream preference) and LS (downstream preference) (Figure 2(c)). These results suggest that for exon definition events the upstream 5′ss, which is proximal across the exon to the upstream 3′ss, is favoured. By contrast, for intron definition events, the 5′ss proximal across the intron to the downstream 3′ss is favoured.

### The influence of exon size on 5′ss selection

It is known that exon size can influence splice site selection [9,10]. To determine the influence of exon size on splice site selection, we compared splice patterns between three different exon size groups, exons smaller than 50 nts, exons between 50–250 nts in length, and exon longer than 250 nts. These cut-offs were chosen based on natural exon size distributions. We then calculated how frequently the major isoform contains the stronger 5′ss for the three different exon size classes (Table 1). When the major and the alternative exons are smaller than 50 nts, splice preference is driven almost exclusively by the stronger splice site score (Table 1). This preference weakens when the usage of the alternative 5′ss generates an exon greater than 50 nts. Thus, differences in exon size contribute to splice site selection, with a preference for generating shorter exons. A similar trend is observed for alternative patterns of major exons within the 50–250 nts range. The selection of alternative exons larger than 250 nts is much less likely to be driven by splice site differences. These data provide evidence that exon size contributes to splice site selection with a preference for defining smaller exons.

**Table 1.** Alternative 5′ss selection and resulting exon length correlation. The table reports how frequently the major isoform contains the stronger splice site when the alternative splice site lies within one of three different exon size classes. ^a,b^Within a column, means without a common superscript differ (p < 0.05) between size categories in each column. ^1^29% preference for the upstream 5′ss. ^2^42% preference for the upstream 5′ss. ^3^44% preference for the upstream 5′ss.

	Exon size generated with major splice site usage			
Exon size generated with minor splice site usage		Ex ≤50 nts	50< Ex ≤250 nts	Ex >250 nts
	Ex ≤50 nts	98%^a1^	86%^a^	100%^a^
	50< Ex ≤250 nts	73%^b^	87%^a2^	88%^a^
	Ex >250 nts	75%^b^	58%^b^	77%^b3^

### Experimental verification of genome-wide computational analysis

**Figure 3.**
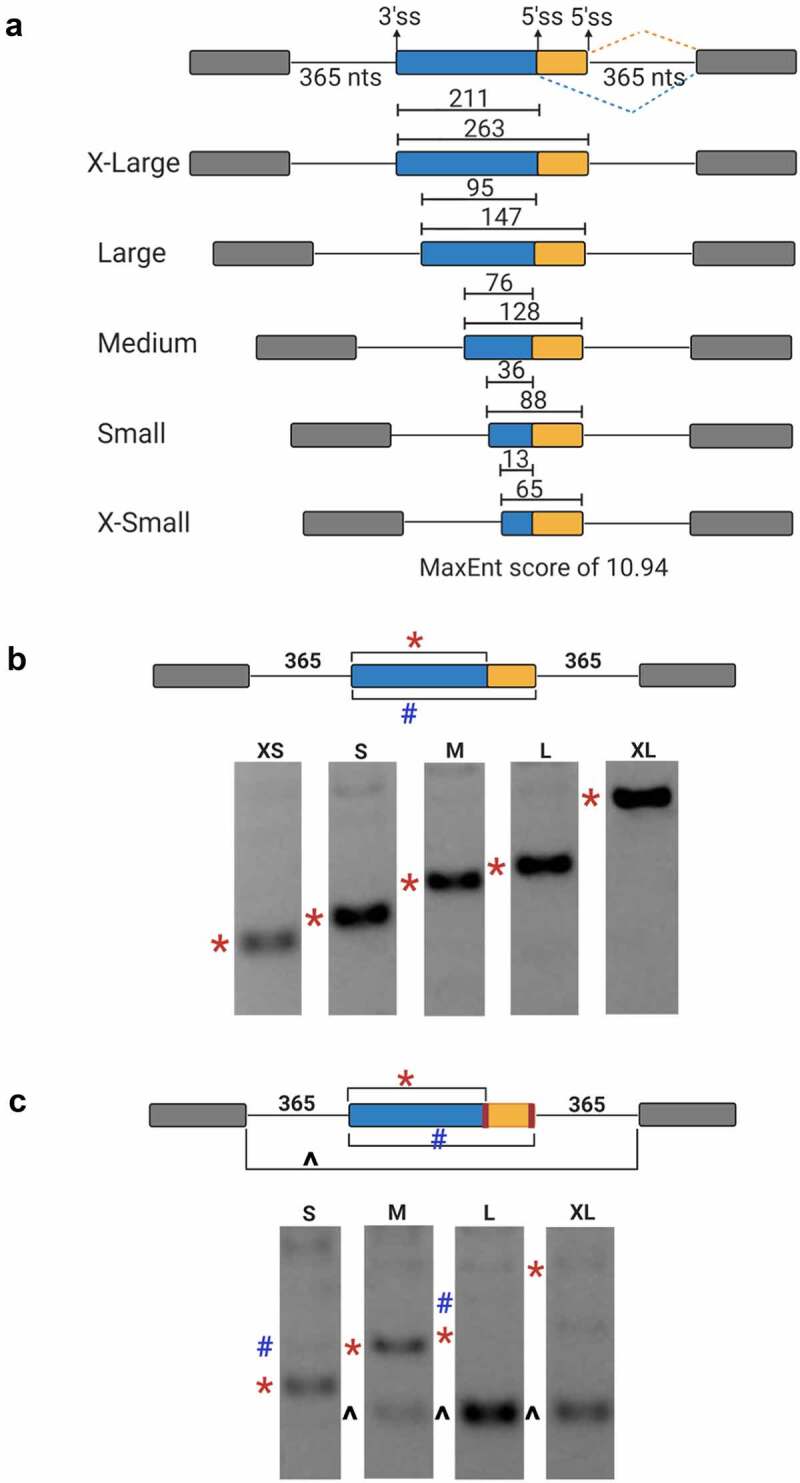
Cross-exon selection of alternative 5′ alternative splice sites. (a) Schematic of exon-defined mini-gene constructs with identical splice site strength (CAG/guaagu, MaxEnt = 10.9) used in transfection experiments. The size of the resulting internal exon is indicated for upstream (blue) and downstream 5′ ss selection. (b) Representative image of ethidium bromide stained agarose gel splicing analysis. Bands denoting upstream (red symbol) or downstream (blue symbol) 5′ss usage are marked to the left of the image. (c) Splicing outcome of minigene constructs with identical but weakened competing 5′ ss (GAG/guguca, MaxEnt = −0.5). Bands denoting upstream 5ʹss usage (red symbol), downstream (blue symbol) 5′ ss usage, or exon skipping (black symbol) are marked to the left of the image.

To test whether the proposed exon-centric proximity rule can be confirmed experimentally, we tested five minigenes that contain an internal exon with two competing 5′ splice sites of identical strength (MaxEnt 10.9, CAG/guaagu) and one 3′splice site with a MaxEnt of 12.56 (ugucccuuuuuuuuccacag/CUG) (Figure 3(a)). All minigenes were designed to be recognized through exon definition (flanking intron size of 365 nts) and differ only in the resulting internal exon size. Cell transfection experiments demonstrated that for all constructs tested the upstream 5′ss was chosen exclusively (Figure 3(b)), consistent with the computational analysis demonstrating that upstream 5′ splices sites are favoured under exon definition. To test if this splice site preference is altered when both competing splice sites are weakened, we mutated both 5′ splice sites to have a MaxEnt score of −0.5 (GAG/guguca). In the larger exon constructs (L and XL), this resulted in preferential internal exon skipping. In the M and S constructs, the upstream 5′ss maintained its preference (Figure 3(c)). These results demonstrate that in an isogenic exon definition context the 5′ss most proximal to the upstream 3′ss is favoured, supporting the computational analysis of an exon definition ‘cross-exon proximity’ preference.

### The influence of intron architecture on 3′ss selection

**Figure 4.**
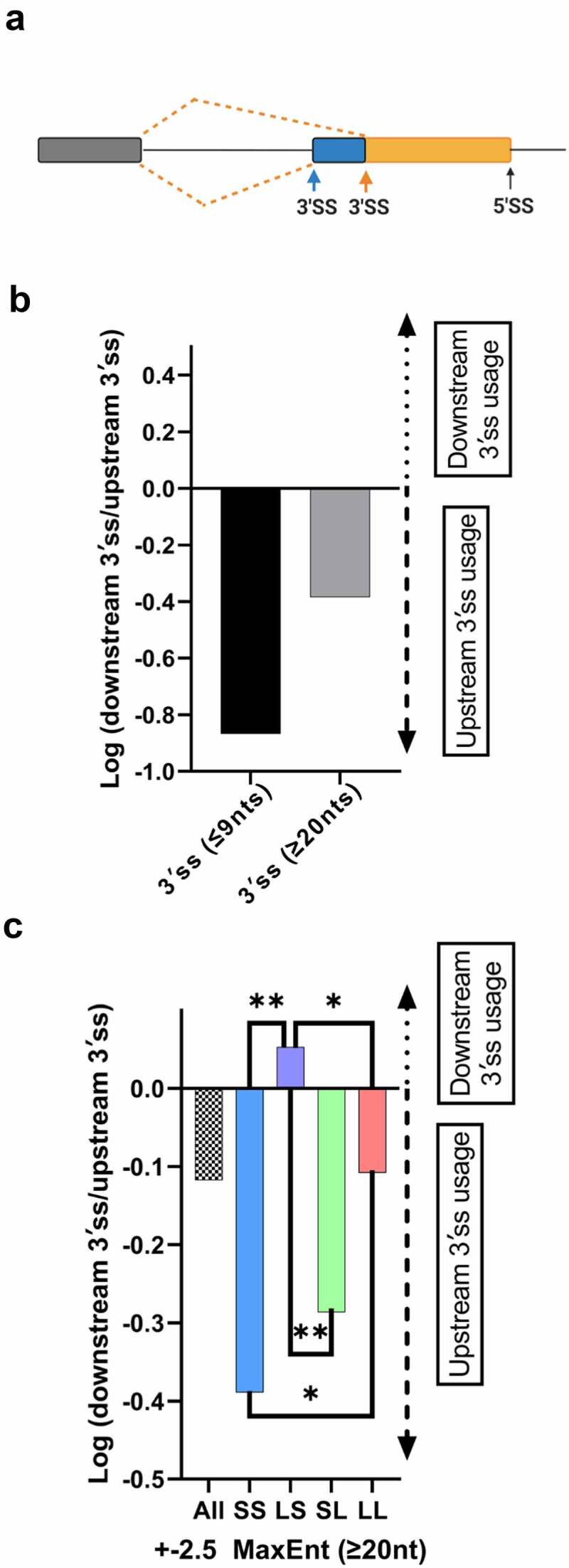
3′ss selection preference for different internal exon categories. (a) Model depicting alternative 3′ss patterns. (b) Bar graph displaying the 3′ss preference for first (>20 nts distance between competing 3'splice sites, 2317 events) or second (<9 nts distance between competing splice sites, 3839 events) step selection. A positive log ratio represents downstream 3′ss preference, a negative log ratio represents upstream 3′ss preference. (c) Bar graph depicting the preference for downstream or upstream 3′ss selection with near equal splice-site strength scores for different internal exon categories (∆ ± 2.5 MaxEntsample size = 69SS, 664 LL, 88 SL, 147 LS). Fisher’s exact test was performed. (b, c), **p < 0.01, * p < 0.05.

To investigate the impact of intron size and splice site strength on 3′ss selection we built a 3′ss dataset analogous to the 5′ss dataset described above (Figure 4(a)). For our analysis, we took into consideration that the 3′ss is recognized during the first and the second steps of splicing. Prior to the first step of splicing, the polypyrimidine tract is bound U2AF, which subsequently recruits U2 snRNP to the branch point. After the first step of splicing, the 3′ splice junction YAG/N is selected before the exons are ligated via a transesterification reaction. It has been demonstrated that competing 3′ splice sites in close proximity (up to 9 nts) are selected during the second step of splicing after identical first step definition [17]. Alternative 3′ splice sites further apart (greater than 12–20 nts) are typically defined during initial splice site recognition using different polypyrimidine tract and branch points. Thus, we split the 3′ss dataset into ‘first step recognition’ (≥ 20 nts apart from one another, 3839 events) and ‘second step recognition’ events (≤ 9 nts apart from one another, 2317 events). Both 3′ss event groups show a preference for upstream 3′ss usage, consistent with the intron-centric proximity rule (Figure 4(b)). This preference is particularly strong for the second step alternative 3′ss events. Filtering to obtain competing 3′ss pairs with comparable strengths and categorizing these events into intron (S/S, 69 events) or exon definition (L/L, 664) events again demonstrated the influence of the intron architecture on 3′ss selection (Figure 4(c)). The strong upstream 3′ss preference observed for intron defined events (S/S) is significantly reduced when splice sites are selected in the exon definition mode (L/L). Consistent with our 5′ss analysis, the hybrid classes (SL, 88 events and LS, 147 events) display more extreme splice site preferences relative to the SS and LL classes, with SL mimicking intron definition and LS mimicking exon definition behaviour.

Together, our transcriptome-wide analyses demonstrate that the mode of splice site selection critically influences splice site choice. For intron definition, splice sites closest across the intron are preferentially selected. Under exon definition, the selection of splice sites closest across the internal exon are favoured. These results suggest that the gene architecture influences alternative splicing by promoting splice site recognition via the intron or exon definition pathway.

## Discussion

The regulation of pre-mRNA splicing is a combinatorial process that is controlled by splice site sequences, cis-regulatory elements binding trans-acting factors, the intron-exon architecture, and RNA secondary structure among other features [16]. Two mechanisms of splice site recognition have been proposed within the broader concept of intron-exon architecture. It has been postulated that under the intron definition splice sites are recognized across the intron, making the intron the initial unit recognized by the spliceosome. In an alternative mode of splice site recognition, splice sites are postulated to be initially recognized across the exon in a process called exon definition. Once the exon is defined as the initial unit of splice site recognition, subsequent structural rearrangements are predicted to recognize and pair the upstream and downstream splice sites across flanking introns [18]. The mechanisms of intron and exon definition have been studied in the field for almost 30 years [9,10,18–23].

Early evidence that the length of introns and exons is important came from size constraints on exon inclusion from minigenes that were transfected in cell culture. Large exons were efficiently spliced when flanked by short introns, consistent with an intron definition mechanism. However, when intron lengths were increased exons were only included efficiently if they were relatively short, less than ~500 nts long. The latter observation suggests that the early spliceosome has a limited ‘wing-span’ when the exon is the unit of initial splice site recognition. Subsequently, biochemical studies demonstrated that intron definition is more efficient and that the rate of splicing for exon defined substrates is considerably slower. This study identified intron length as the primary determinant in the mode of splice site recognition employed by the early spliceosome and placed the transition from intron definition to exon-definition at the point when flanking introns become longer than 200–250 nts [10].

Another classical study used *in vitro* splicing assays to demonstrate that alternative splice site choice is influenced by the proximity between the pairing splice sites. When two splice sites are in competition, the splice site proximal to the intron is preferred. As a result, this proximity bias induces the preferential excision of the smaller intron [11]. This study and the pioneering study from Sterner and Berget when analysed together suggest that in the context of splice site competition, selection of proximal splice sites across an intron may allow the intron to be recognized through intron definition, while the selection of the distal splice site may lead to a larger unit of initial splice site recognition that may change the mode of splice site recognition all together [9,11]. In broader terms, the findings by Reed and Maniatis [11] indicated that perhaps proximity across the initial unit of splice site recognition would drive splice site selection and influence alternative splicing. We set out to determine whether the proximity of splice sites across the proposed initial unit of splice site recognition may provide genome-wide evidence for the two modes of splice site recognition and elucidate their roles in alternative splicing.

**Figure 5.**
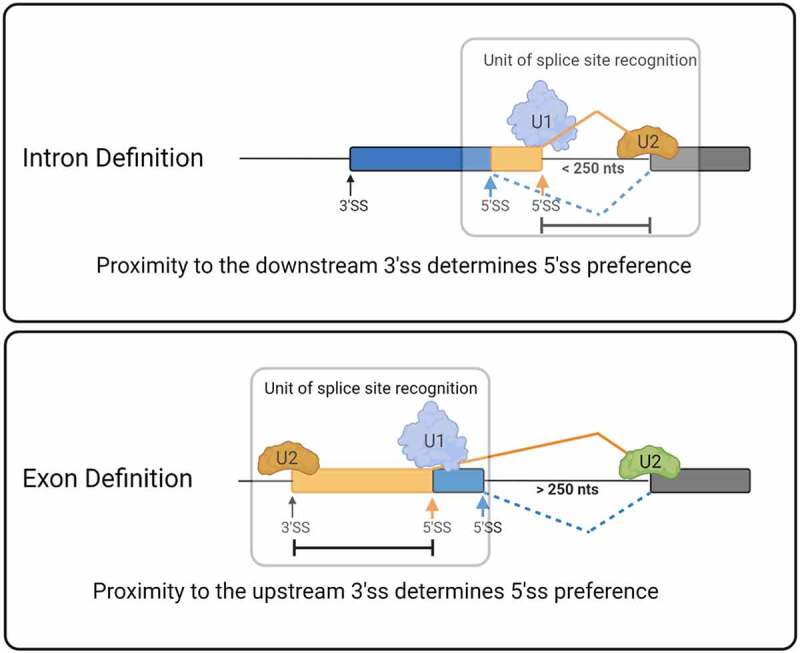
Unifying model for the influence of splice site proximity in alternative exon definition. Depending on the size of flanking introns the splice sites of internal exons are initially recognized across the intron (top – intron definition) or across the exon (bottom – exon definition). In both scenarios, the 5′ and 3′ splice sites closest to each other across the unit of initial splice site recognition are preferentially selected. Thus, in intron definition 5′ and 3′ splice sites across the intron are preferentially selected. In exon definition, 5′ and 3′ splice sites across the exon are preferentially selected.

Our analysis of the alternative splicing events captured in ALTssDB permitted the derivation of several important conclusions. First, the intron-centric proximity rule observed by Reed and Maniatis is maintained within the context of the intron definition mode of splice site recognition [11]. In the context of exon definition, we observe an exon-centric proximity rule, where the proximity between 5′ and 3′ splice sites across the exon dictates splice site preference. Alternative exons subject to the intron-centric proximity rule undergo removal of the smaller intron and selection of the larger exon. Conversely, alternative exons subject to the exon-centric proximity rule undergo removal of the larger intron and selection of the smaller exon. Initially, these observations may appear inconsistent with each other, yet they highlight a commonality of spliceosomal assembly across the smallest unit of initial splice site recognition. For the intron definition mode of splice site recognition this unit is the intron, meaning the spliceosome assembles around the 5′ and 3′ splice sites that define the intron to be excised (Figure 5, top cartoon). For the exon definition mode of splice site recognition, the unit of recognition is the exon, meaning that initial splice site recognition by the spliceosome occurs across the exon (Figure 5, bottom cartoon). In both modes of splice site recognition, a preference for splice site selection that promotes the definition of the smaller initial recognition unit (as defined by the number of nucleotides) is observed. Thus, the proximity of 5′ and 3′ splice sites within the unit of initial recognition determines preferential splice site selection (Figure 5). We therefore conclude that an additional mechanism of alternative splicing can be the proximity of splice sites across the initial unit of definition.

Since the initial concepts of intron and exon definition were introduced, generating supporting evidence for the existence of these two proposed modes of splice site recognition has been challenging. Initial studies were limited to select cases where insights were gained from transfected designer minigenes or *in vitro* transcribed RNAs spliced using the nuclear extract system [9,10,19,21–23]. These studies, while mechanistically enlightening, could not be extrapolated to the entire transcriptome.

Recent analyses of *in vivo* splicing kinetics offer more comprehensive insights into the mechanisms of exon recognition. These studies lend support to the notion that exon and intron definition events display different global splicing kinetics. They also raise questions about the generality of exon definition and intron definition [24–26]. A single molecule intron tracking technique was used to determine the amount of splicing as a function of RNA polymerase II position along the gene. This technique and an orthogonal nanopore-based variation found splicing rates to be strikingly fast in *Saccharomyces cerevisiae* [25] demonstrating that 50% of splicing can be completed 1.4 seconds after 3′ss synthesis for the genes studied. The onset of splicing for a subset of the analysed genes was detected only 26 nucleotides after transcription of the 3′ss. The observation that splicing can be completed before the entire exon is transcribed is consistent with an intron definition mechanism in *Saccharomyces cerevisiae*, but begs the question is exon definition possible in lower eukaryote? The average *Saccharomyces cerevisiae* exon is ~1400 bases suggesting that exon definition would be highly unlikely for those genes where splicing rates were calculated to occur on the order of several seconds [27]. However, a recently proposed unifying model provides evidence for exon definition in *Saccharomyces cerevisiae* [28]. Electron microscopy analyses suggest that the splicing factor Prp40 can bridge the 5ʹss bound U1 snRNP and branch point sequence bound BBP/Mud2 (SF1/U2AF65 homologs) either across the intron or across the exon to define E-complex. Structural evidence for exon definition in *Saccharomyces cerevisiae* was supported by genetic and biochemical analysis, which included the circularization of single exon constructs in yeast splicing extracts. The latter study provides strong structural, biochemical, and *in vivo* evidence for exon definition, even in *Saccharomyces cerevisiae*, where most splice sites would be expected to be recognized through intron definition [28].

Regarding the intron-exon architecture of higher eukaryotes, ligation of 3’ adapters and long read nanopore sequencing of nascent RNA were used to determine the splicing rates in Drosophila and human cells [26]. The nano-COP method determined that the majority of splicing in Drosophila occurs within 2 kilobases, once the 3ʹss has been transcribed. This is in contrast to human cells where the majority of splicing is completed ~4 kilobases past the 3ʹss [26]. The rate of splicing calculated from nano-COP is consistent with previous t_1/2_ measurements that are ~2 minutes for Drosophila and 7–14 minutes for mammalian cells [24,29–31]. Interestingly, nano-COP found that Drosophila introns less than 100 nts in length were spliced more quickly than introns greater than 300 nts, suggesting that intron definition is more efficient than exon definition [26]. These results are supported by an earlier study that used progressive metabolic labelling and also found a local maximum of splicing rates for introns that were 60–70 nts long [24]. However, the latter study also found that a subset of very long introns (>2944 nts) was spliced even more quickly with a t_1/2_ of ~1.5 minutes suggesting gene level and pathway-specific splicing programmes may have evolved to utilize the rapid splicing that very long exon-defined introns undergo. Taken together these kinetic measurements suggest that while exon definition is broadly less efficient and intron definition is broadly more efficient as was first shown by Fox-Walsh and Hertel [10], exceptions do exist.

Recent investigations provide further support that both intron definition and exon definition occur *in vivo* [32,33]. However, these studies present evidence that the mechanism by which splice sites are initially recognized is dictated by the difference in GC content, referred to as GC differential, between the exon and the flanking introns. Specifically, two architectures are described, referred to as the ‘differential architecture’ and the ‘leveled architecture’ [32,33]. ‘Differential architecture’ exons have a low GC content, and their flanking introns have an even lower GC content. 'The ‘leveled architecture’ exons are characterized by a high GC content, less difference in the GC content of flanking introns and short introns. The former class of exons was demonstrated to be localized to the nuclear periphery and recognized through exon definition while the latter was shown to be localized to the nuclear centre and recognized through intron definition. Altering the GC content between exon and the downstream intron can be used to alter the mode splice site recognition, without changing the length of the intron. These observations suggest that intron length may not be the determining factor in the mode of splice site recognition for exon definition [32,33]. It will be important for future exon definition studies to consider the GC content across the exon and flanking introns. For example, a recent analysis of high-throughput mutagenesis data for an alternatively spliced exon in the proto-oncogene *RON* demonstrated that the alternatively spliced exon is recognized through exon definition, even though it is flanked by short introns on either side (87 and 80 nts) [34]. Thus, splice sites of short introns can be recognized through exon definition, perhaps because the unique GC content that typifies exon definition splice sites.

Finally, a recent transcriptome-wide study demonstrated that introns that undergo efficient co-transcriptional splicing have sharp structural transitions across the intron-exon boundary [35]. These introns display a peak of RNA structure downstream of the 5’ss and upstream of the 3’ss. Furthermore, some introns displayed enhanced co-transcriptional splicing under conditions where the elongation rate of RNA polymerase II was slowed down genome-wide, a process that promotes increased RNA folding. The latter group of introns had significantly steeper structural transitions when transcription was slow [35]. GC content is an indicator of the potential to form RNA secondary structures [36]. Thus, it may be the case that the differential architecture associated with exon definition is driven partially by the propensity for RNA secondary structure formation that can help delineate the intron-exon boundary.

We set out to determine the degree of agreement between the intron length-dependent definitions of ‘intron-defined’ and ‘exon-defined’ splice sites with the ‘leveled’ and ‘differential’ architecture. We calculated GC content differentials between the LL and SS architectural classes of alternatively spliced 5’ and 3ʹss exons. Remarkably, the intron length-defined LL and SS categories closely resemble the ‘differential’ and ‘leveled’ architectures respectively [32,33] (Supplemental Figure 2). Thus, the GC content, as defined the Amit et al. [32], of long introns (>250 nts) differs significantly from the GC content of short introns (<250 nts), suggesting that GC content or intron size definitions are comparable approaches to define exon and intron definition modes of splice site recognition. This notion is supported by evolutionary analyses that show the emergence of a distinct differential GC architecture as intron lengths increased through vertebrate evolution [37]. Thus, the emergence of the ‘differential architecture’ may be a co-evolutionary adaptation to define exons in the context of expanding introns. To evaluate whether the use of proximal or distal splice sites changes ‘leveled’ and ‘differential’ architecture designations, we calculated GC content for alternatively spliced exons captured by ALTssDB. Interestingly, the resulting GC differential does not change significantly (Supplemental Figure 2), suggesting that alternative splice site selection is not dependent on differential GC content but contingent on defining the smallest unit of initial recognition.

Collectively, the results of our transcriptome-wide analysis of alternative splice site usage provide evidence that exon definition and intron definition do occur transcriptome-wide. When exons are flanked by long introns, the spliceosome tends to favour splice sites located internally within the exon being defined. By contrast, the spliceosome tends to move into the intron for splice site definition for exons flanked by short introns. These observations suggest that the spliceosome can define the exon and the intron independently.

Our computational analysis of alternative 3′ss events permitted an evaluation of alternative 3′ss selection in the context of first or second step recognition. Initial 3′ss selection is mainly driven by the strength of the polypyrimidine tract and the presence of a consensus branch point. Upon recruitment of U2 snRNP to the branch point and tri-snRNP incorporation, the first step of the splicing reaction is initiated without engaging the 3′ss junction. After spliceosome rearrangements, the 3′ss junction is selected during the second step of splicing as the spliceosome aligns the AG/N intron/exon junction into the active site. It is well established that competing 3′AGs in close proximity (less than 9 nts) use the same upstream polypyrimidine tract and branch point and that their selection is directed during the second step of splicing [17]. Interestingly, our analysis of alternative 3′ss selection in close proximity demonstrated that the upstream AG/N junction is almost exclusively chosen over the downstream AG/N. Thus, it appears that aligning the closest AG/N 3′ss junction is the default pathway of second step splice junction selection (Figure 4(b)).

The intron-exon architecture of genes is a major driver of splice site selection. Since the initial postulation of these two modes of splice site recognition, various forms of evidence have been presented, often in form of kinetic principles supporting intron or exon definition. However, measurements of splicing rates as a function of intron length do not constitute direct evidence of alternative spliceosomal assembly pathways. The ability of yeast E-complex to assemble across the intron or the exon is perhaps the strongest evidence yet for exon definition. Our study provides support for exon definition by demonstrating the spliceosome favours internal splice sites within exons when the splice site strengths of competing sites are comparable. This suggests that the exon is being defined and not the intron. This study provides a unifying model for splice site selection, whereby the spliceosome assembles across the smallest unit of initial splice site recognition. In the case of intron definition, this entails removal of smaller introns and inclusion of larger exons. Indeed, studying the evolutionary trends in intron-exon architecture, lower eukaryotes tend to have larger exons and smaller introns. Upon intron expansion and a gradual shift towards exon defined gene architecture, the initial unit of splice site recognition often tends to be the exons. This may be due to the increased number of decoy splice signals associated with larger genome sizes. It would therefore be expected that the smaller exons would be favoured in higher eukaryotes. This trend is also broadly observed from yeast to humans. It is possible that the exon-centric proximity rule is an evolutionary adaptation to accurately recognize exons surrounded by long stretches of intronic sequence. Our results not only provide *in vivo* and transcriptome-wide evidence for exon definition, they also demonstrate that exon and intron definition influence alternative splicing in the context of alternative 5’ or 3’ splice site competition.

## Methods

### Construction of ALTssDB

ALTssDB was created using EST data from the Human Exon splicing Events (HEXEvent) database [13]. HEXEvent contains information regarding the location of competing splice sites, the resulting exon sizes, alternative splice site usage levels and the gene associated with each mRNA. The HEXEvent data was filtered to obtain a dataset comprising of only pairs of competing 5’ and 3’ splice sites separately. This database was subsequently modified to include splice site junction information and splice site strength scores using MaxEntScan [6]. Although other approaches exist to evaluate the strength of 5 splice sites [38,39], MaxEntScan is the preferred tool as it also permits comparable splice site score derivation for 3 splice sites. Using an R script and IntronDB dataset, (a database detailing eukaryotic intron features) flanking intron lengths were added to the database [14]. Alternative splicing events were further filtered to include only events that have 10 or more EST counts. The data was filtered into four categories according to intron length and included: both flanking introns around the exon of interest being short (<250 nts, SS), both flanking introns being long (>250 nts, LL), the upstream intron being short and downstream intron being long (SL) or the upstream intron being long and the downstream intron being short (LS). ALTssDB does not differentiate between canonical U2 introns and U12-type introns. Given their rarity and limited involvement in alternative splicing beyond intron retention, it is anticipated that U12-type introns are not well represented in ALTssDB [40].

ALTssDB does not distinguish between isoforms that originate from a tissue specific splice switch or disease comparison. It lists all known splice patterns for a particular exon, independent of origin. EST data was used to build AltssDB to obtain high enough numbers of alternative splice site choices within the human genome to carry out all analyses. While datasets for tissue-specific splicing are available, the quantity of significant alternative splice site events is limiting.

### Plasmid design

Five minigene constructs were designed containing three exons and two introns. The plasmid design was based primarily on previously validated constructs used to study splice site strength [7]. The internal exon was designed to contain two functional competing 5’ splice sites (CAG/guaagu), with equal MaxEnt scores MES of 10.9, separated by 52 nucleotides. The sequence preceding the upstream 5’ splice site was progressively shortened (Figure 1(b)). Additional constructs were created where the MES of both competing 5’ splice sites were changed from 10.9 to −0.5 (GAG/guguca) for S, M, L, and XL plasmid. Lastly, the upstream 5’ splice sites were changed from MaxEnt = 10.9 to MaxEnt = −5.2 (UCG/gucgau) for the S, M, and XL to show that the downstream 5ʹss is viable (Supplementary Figure. 1).

### Cloning protocols to change splice site strength sequences

To linearize the plasmids, 10 nanograms (ng) of plasmid DNA obtained by midiprep was amplified using divergent primers. PCR reactions were carried out with NEB® Phusion® polymerase in 50 µL according to NEB protocols. DH5α E.coli midiprep derived plasmids in the PCR reaction were digested with 40 units of DpnI according to NEB protocols. Plasmids were purified with Zymo DNA clean and concentrator™ kit and DNA concentrations were obtained using a nanodrop 2000 instrument. For each construct, 0.03 picomoles of linearized plasmid DNA was mixed with a 10X molar ratio of phosphorylated double stranded DNA inserts, purchased from IDT, in 20 µL ligation reactions. Synthetic inserts were cloned into linearized vectors using T4 ligase according to the standard NEB protocol and 10 µL of the ligation reaction was transformed using in house DH5α E.coli cells. Colonies were screened using PCR to detect the correct size insert. Colonies with the correct size insert were grown from 3 mL cultures to 20 mL cultures and underwent midiprep DNA extraction. Plasmid DNA from each colony was sequenced to ensure the correct orientation of inserts.

### Cell transfections and RT-PCR Analysis

Transfection experiments were performed in triplicate using HeLa cells. 1 mL of 0.1 × 10^6^ cells/mL was plated into each well of 12 well plates. Cell confluency was checked 24 hours later and 1 µg of plasmid DNA was transfected according to Bioland Scientific’s BioT protocol. Cells were harvested 48 hours post-transfection. Each well was washed two times with phosphate buffered saline (PBS) and subsequently RNA was extracted with the standard Trizol™ protocol. RNA pellets were resuspended in 50 µL water and put through ZYMO RNA Clean and Concentrator™ columns. Sample volumes were adjusted to 80 µL, yielding RNA concentrations of ≤200 ng/µL. DNase digestion was performed with Turbo™ DNase (Ambion®) according to Ambion’s protocol in 100 µL reactions. RNA was subsequently extracted with 100 µL phenol: chloroform and the aqueous phase was put through ZYMO RNA Clean and Concentrator™ columns. DNase digested and purified RNA samples were resuspended in 25 µL. A nanodrop 2000 instrument was used to obtain RNA concentrations. Reverse transcription reactions were carried out in 20 µL using 250 ng of total RNA and 200 ng of OligodT18 primer according to SuperScript™ III protocol. PCR primers are as followed: first exon forward primer (5′cgttcgtcctcactctcttc3′) and third exon reverse primer (5′agatccccaaaggactcaaaga3′). PCR primers were designed that bound the flanking exons and thus would detect upstream, proximal or downstream, distal 5’ splice site usage. PCR reactions contained 5 µL cDNA (10% vol:vol), 0.2 mM dNTPs, 0.2 µM of each primer, 1.5 mM MgCl2 and 0.25 units taq polymerase (Apex Bioresearch). Semi-quantitative PCR using long extension times to limit PCR product size bias was carried out to demonstrate that the ratio of upstream and downstream splice site usage, or the alternative exon skipping pattern, remained constant throughout the dynamic linear range of the amplification reaction (data not shown). Based on these results 25 cycles of PCR were performed for each sample and 5 µL was subsequently loaded onto a 2% agarose gel and stained with ethidium bromide. Agarose gels were run at 150 V for 1 hour in 1X Tris-Borate EDTA (TBE).

### Calculating splice site selection preference

5′ss selection preference was determined by calculating the log ratio of the number of splice site events preferring the downstream 5′ss over the upstream 5′ss. 3′ splice site selection preference was determined by calculating the log ratio of the number of splice site events preferring the downstream 3′ss over the upstream 3′ss.

## Supplementary Material

Supplemental Material

## Data Availability

The data that support the findings of this study are openly available in Dryad at (https://datadryad.org/stash/share/kVSpiiUJbVhjuHysLWLgG38JjgYJy_UdcpODKw6DXs8).
