# Supplementary material for: Splice site proximity influences alternative exon definition
Source: RNA Biol. 2022 Jun 19;19(1):829–40. doi: 10.1080/15476286.2022.2089478 (PMC9225289; doi:10.1080/15476286.2022.2089478)
Supplement: Supplemental Material [file KRNB_A_2089478_SM1609.pdf]

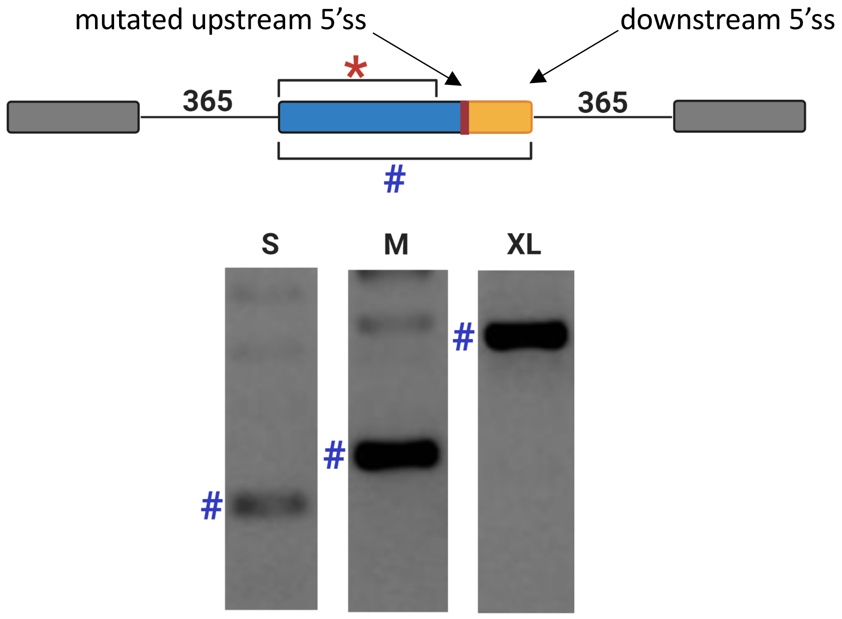

Supplementary Figure 1. The downstream 5′ splice site is a functional splice site. Cartoon and representative image of the splicing outcome of minigenes containing a mutated and non-functional upstream 5′ splice site (MaxEnt = -5.2, UCG/gucgau). Bands denoting downstream (purple symbol) or upstream (red symbol) 5′ splice site usage are marked to the left. Spliced products were separated using ethidium bromide-stained agarose gels.

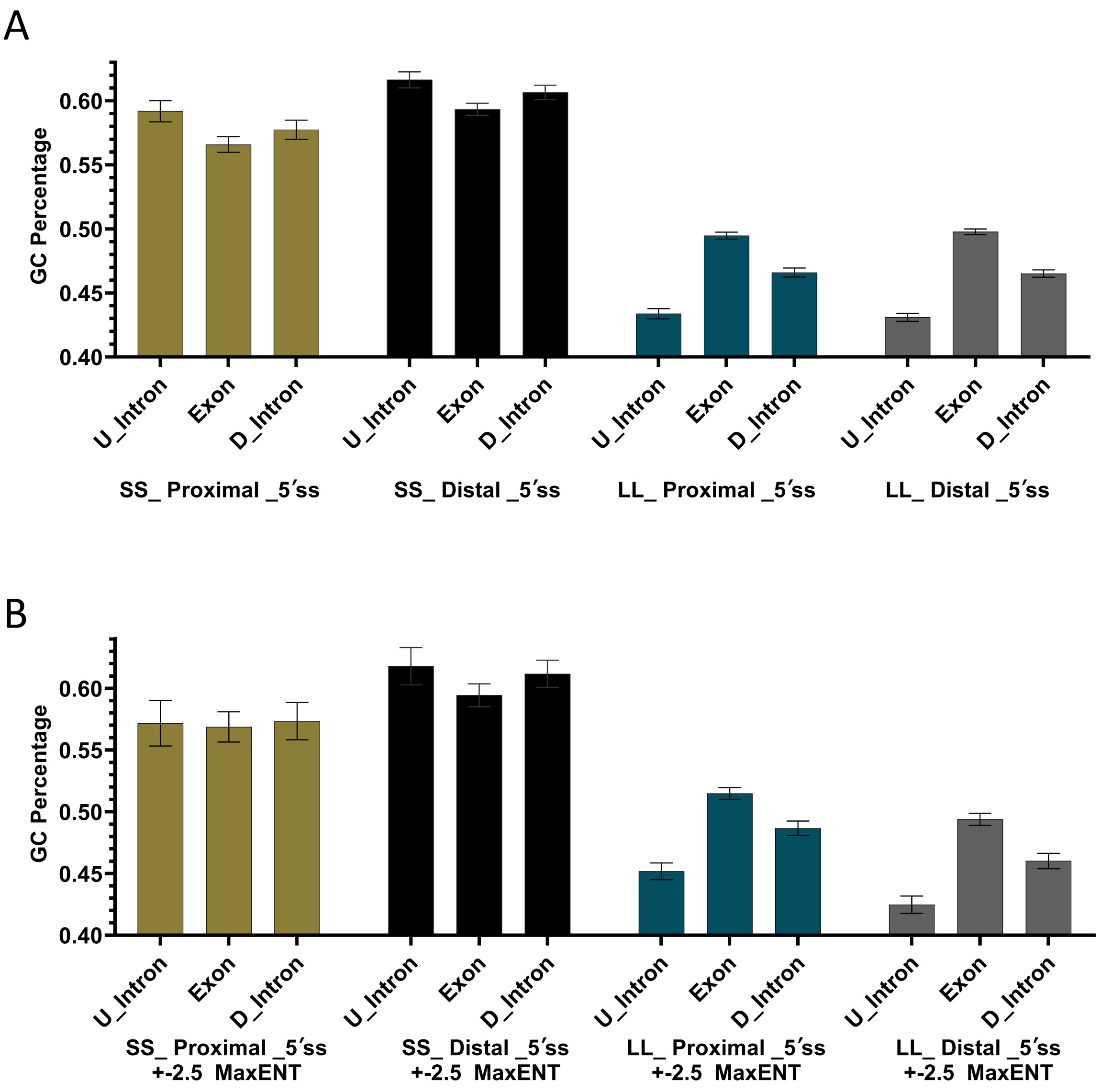

Supplementary Figure 2. GC content distribution for intron definition splice sites (SS) and exon definition splice sites (LL) based on the intron length-dependent classification used herein (SS<250 nts, LL>250 nts) (A) The data summarized in the graphs represent all alternatively spliced SS and LL 5′ splice sites captured by ALTssDB. The GC content as defined by Amit et al [32] is displayed for intron definition (SS, forest green and black) and exon definition (LL, crimson blue and grey) events. GC content for each architectural class was computed for the exon and intron when the exon/intron junction is defined by the major 5′ splice site (SS, forest green and LL, crimson blue) or the minor 5′ splice site (SS, black and LL, grey) of the alternatively spliced exon. The comparison between SS and LL architectural definitions shows a striking GC content difference as was observed by Amit et al [32]. The profiles do not change significantly when the major or the minor 5′ splice site is used. (B) The data summarized in the graphs represent alternatively spliced SS and LL 5′ splice sites with near equal splice site scores (+/-2.5 MaxEnt). Bar graph definitions are as described in (A). Filtering the alternative 5′ splice site analysis by near equal splice site scores does not significantly change the outcome. The comparison between SS and LL architectural definitions highlights GC content difference as was observed by Amit et al [32] and the profiles do not change when the major or the minor 5′ splice site is used.
